# How I do it: transapical cannulation for acute type-A aortic dissection

**DOI:** 10.1186/1749-8090-3-4

**Published:** 2008-01-29

**Authors:** Andrzej W Sosnowski, Rajwinder S Jutley, Nicola Masala, Christos Alexiou, Justiaan Swanevelder

**Affiliations:** 1 Department of Cardiac Surgery, Glenfield Hospital, Leicester, UK; 2 Department of Cardiac Anaesthesia, Glenfield Hospital, Leicester, UK

## Abstract

Aortic dissection is the most frequently diagnosed lethal disease of the aorta. Half of all patients with acute type-A aortic dissection die within 48 hours of presentation. There is still debate as to the optimal site of arterial cannulation for establishing cardiopulmonary bypass in patients with type-A aortic dissection.

Femoral artery cannulation with retrograde perfusion is the most common method but because of the risk of malperfusion of vital organs and atheroembolism related to it different sites such as the axillary artery, the innominate artery and the aortic arch are used. Cannulation of these sites is not without risks of atheroembolism, neurovascular complications and can be time consuming. Another yet to be popularised option is the transapical aortic cannulation (TAC) described in this article. TAC consists of the insertion of the arterial cannula through the apex of the left ventricle and the aortic valve to lie in the sinus of Valsalva. Trans-oesophageal guidance is necessary to ensure correct placement of the cannula.

TAC is an excellent method of establishing cardiopulmonary bypass as it is quick, provides a more physiological method of delivering antegrade arterial flow and is the only method to assure perfusion of the true lumen.

## Background

Aortic dissection is the most frequently diagnosed lethal disease of the aorta. Half of all patients diagnosed with acute type-A aortic dissection die within 48 hours of presentation. The principal aim of surgery in type-A aortic dissection is to prevent aortic rupture and further dissection into the aortic valve and coronary ostia.

The most popular approach to establishing arterial return for cardiopulmonary bypass (CPB) in type-A aortic dissection is femoral cannulation with retrograde perfusion of the aorta. Although this technique has been used since 1950s, it is not without its problems. The retrograde perfusion of the aorta has a potential risk of cerebral embolisation of atheromatous debris and extension of the dissection flap. The technique may also result in intraoperative malperfusion of aortic branch vessels. The alternative sites for arterial cannulation which also allow antergrade perfusion of the aorta include the aortic arch, axillary artery and the innominate artery [1,2].

However, these approaches are not strictly antegrade as there is still retrograde flow down the cannulated vessels to the aortic arch.

There remains a useful and yet to be popularised option of aortic cannulation through the apex of the left ventricle and aortic valve. This transapical or transventricular technique was described by Zwart as a part of a left ventricular support system 35 years ago [3]. In 1985 Golding described the successful application of the technique for a coronary bypass operation in the presence of severely atherosclerotic ascending aorta [4]. It was not until 1991 that Robicsek reported its use for acute aortic dissection [5]. More recently, Wada *et al *published their large series with excellent clinical results. In over 130 patients they had no malperfusion events with a low mortality of less than 20% [6]. Despite these favourable reports the technique has yet to be popularised by the surgeons or properly evaluated for reasons that remain unclear. In this paper we describe our technique of transapical aortic cannulation for acute type-A dissection.

### Patient selection for surgery

The transapical approach is useful in all patients with acute type-A dissection irrespective of the extent of the dissection flap. It is particularly useful in complicated cases where other conventional methods of cannulation would have been precluded. For example, in our experience we have successfully used the technique in patients where the dissection flap involved the subclavian and femoral vessels. It was also particularly effective in one unusual case of recurrent dissection of the ascending aorta which presented several years following the first successful repair. In this case, the arterial return was established using the transapical approach through a small left thoracotomy.

### Surgical technique

#### Patient anaesthesia

The operation is performed under general anaesthesia with the usual cardio-respiratory monitoring equipment. Additional monitoring is necessary for deep circulatory hypothermic arrest (DCHA) and pulmonary artery wedge pressure (PAWP) monitoring. If the patient is haemodynamically unstable the PAWP line is deferred until after the surgery. We place a trans-oesophageal echocardiogram (TOE) probe in all cases, to guide the transapical arterial cannula through the aortic valve before CPB is instituted. The TOE also allows us to inspect the aortic valve and assess feasibility for repair as well as assess the true lumen at the level of the ascending aorta.

#### Patient setup

There are no additional setup procedures necessary prior to surgery. As with other approaches for acute type-A aortic dissection we prepare and drape the patient to include the legs in case bypass grafting using saphenous vein is necessary. Both groins are exposed but not cannulated. All perfusion lines are primed and prepared prior to surgery with the perfusionist ready to crash onto CPB should the patient suddenly decompensate.

#### Surgical incisions and cardiopulmonary bypass conduct

**Figure 1:**
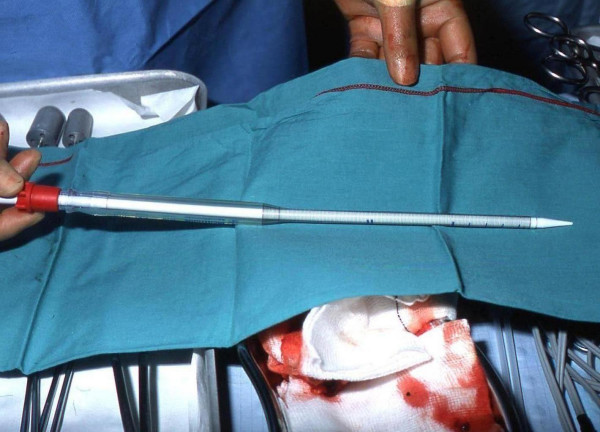
The DLP^® ^22-French EOPA arterial cannula. It measures 30.5 cm and is wire-reinforced to prevent kinking.

The patient is fully heparinised (300 units/kg) if there is any haemodynamic instability prior to surgery. Otherwise, it is administered after the chest is opened through a median sternotomy incision and the heart exposed using the conventional pericardiotomy approach. Once any pericardial effusion is suctioned away, we place a purse string in the right atrial appendage using 3-0 Tycron sutures (Tyco Healthcare, Mansfield, MA). A 2-stage cannula is then inserted for venous drainage and connected in readiness for CPB. There is no custom-made cannula for transapical arterial cannulation. We use a DLP^® ^22-French EOPA arterial cannula (Medtronic, Minneapolis, MN) as shown in Figure 1. This 30.5 cm length cannula is inserted over a flexible introducer and is of single-piece construction. It is also wire reinforced allowing considerable flexibility without kinking.

**Figure 2:**
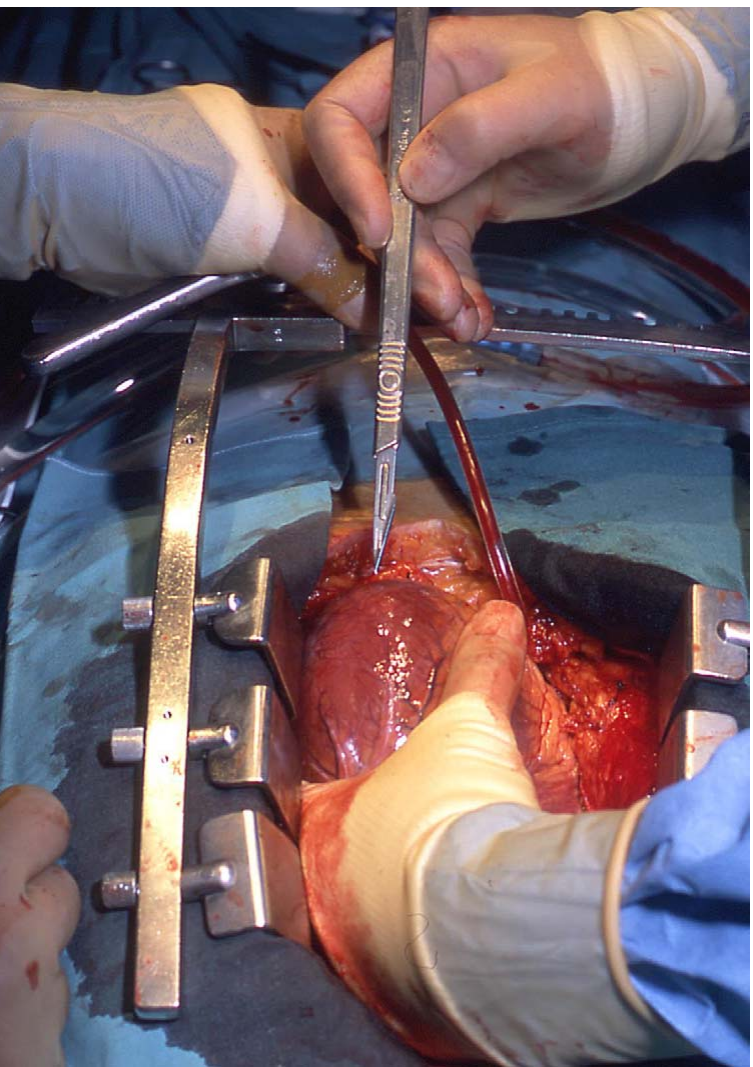
A stab incision is made in the left ventricular apex using a No 11 bladed knife.

**Figure 3:**
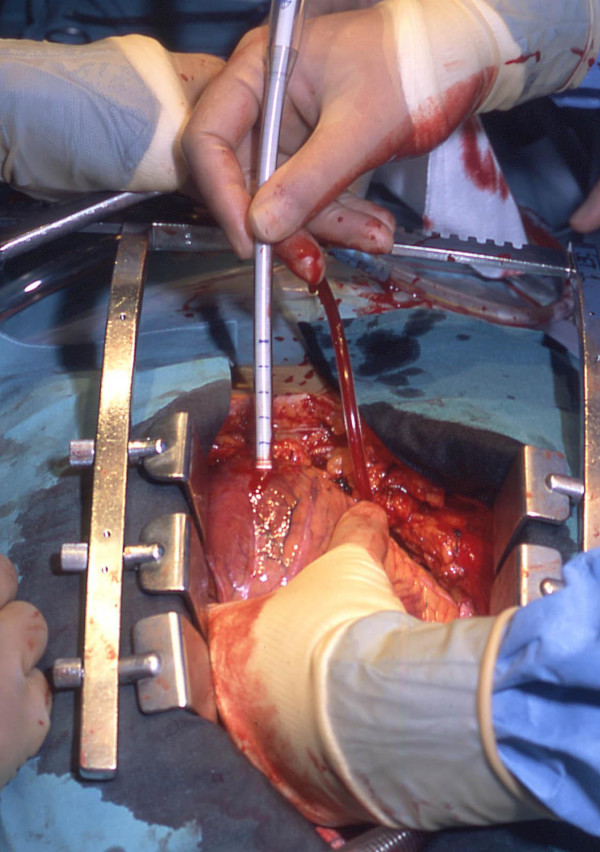
The cannula inserted so that the tip lies beyond the aortic valve.

**Figure 4:**
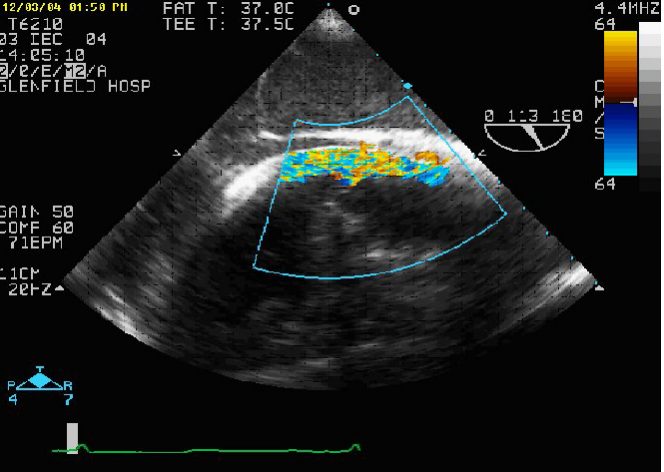
This TOE shows the tip of the cannula lying at the level of the sinus of Valsalva with perfusion of the true lumen.

To insert the arterial cannula we lift the heart and make a small stab incision (typically less than 5 mm in length) in the apex using a No 11 bladed knife (Figure 2). It is important not to traverse the myocardium with the incision but to breach the epicardium. In this way, the ventricular muscle fibres are split along their length as the cannula is introduced. We believe that this makes the closure of the apex more haemostatic and has in fact obviated the use of pledgeted closure in our experience [7]. The cannula tip is then inserted (Figure 3) and guided to lie across the aortic valve in the sinus of Valsalva under TOE guidance (Figure 4). Although location of the cannula tip in the sinus of Valsalva is preferred, it is not necessary. In some cases of gross cardiomegaly the cannula used was too short to traverse the aortic valve such that the tip was located in the left ventricular outflow tract (LVOT). There was no compromise in flow rates on CPB or any adverse clinical outcome with the location of the cannula in the LVOT.

Once the cannula is located CPB is commenced with cooling to 17°C. If the heart distends it is easily vented through a small stab incision in the pulmonary artery assisted with intermittent squeezing of the left ventricle. In our experience distension is rarely problematic. We have found that even in the presence of severe aortic regurgitation due to acute dissection the aortic leaflets co-apt against the cannula wall reducing any incompetence.

**Figure 5:**
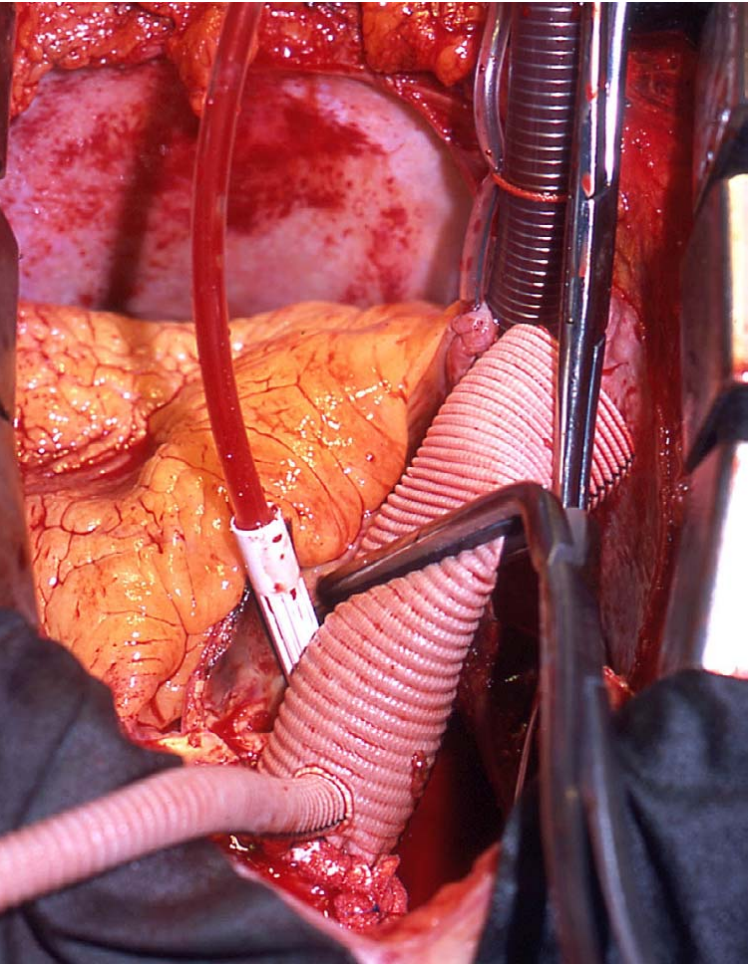
Once the distal anastomosis is completed CPB is recommenced through the side arm of the graft. The distal part of the operation can then be performed.

Once deep hypothermic circulatory arrest is established at 17°C the aortic cannula is removed and the aorta transected below the takeoff of the innominate artery. The arch is inspected to exclude further entry sites. An end-to-end distal anastomosis is then performed performed using a 3-0 monofilament polypropylene suture reinforced with a felt strip. We prefer a Dacron graft (Gelweave, Vascutek Inc, Ann Arbor, MI) with an 8 mm sidearm. Once the distal anastomosis is complete the graft is de-aired by re-starting slow controlled retrograde perfusion via the venous cannula. CPB is then re-instituted through the side arm of the graft and re-warming commenced as seen in Figure 5. During re-warming a conventional vent is inserted into the apical cannulation site. Attention is then paid to the aortic valve and repair or replacement performed as necessary. In case of replacement we prefer a stentless bioprosthesis such as Elan Root (Vascutek Ltd., Glasgow, UK) which allows an end-to-end anastomosis with the Dacron graft.

Once the heart is adequately de-aired the apical vent is removed and the cannulation site closed with a horizontal mattress 4-0 polypropylene suture with an over-and-over running stitch. We do not use pledgets for a reinforced closure.

#### Post-operative care

We routinely perform CT-scans and trans-thoracic echocardiography in all patients prior to discharge and on regular intervals following discharge as part of their surveillance program.

### Results of the transapical technique

By far the largest series of transapical cannulation for type-A dissection consisted of 138 patients reported recently by Wada *et al *[6]. Prior to this report most publications were simple case reports or small series. Although most readily support the use of transapical cannulation as the technique of choice for type-A dissection, the modest patient numbers may explain the low uptake of the technique. In their extensive experience Wada *et al *encountered no malperfusion events or the need to convert to another cannulation technique. Their overall mortality was less than 20% with no deaths or morbidity related to the cannulation technique. The overall rate of cerebrovascular accidents was impressively low at 5.8%. These observations by Wada are reflected in our smaller series. Moreover, we have identified no problems such as pseudo-aneurysm formation with the cannulation site on surveillance CT scanning.

### Advantages of the transapical technique

The transapical cannulation technique offers several advantages over the traditional femoral or axillary approach. A major advantage of the transapical cannulation technique is that it is quicker to insert than other conventional methods as no purse-strings or additional dissection is required. Establishing arterial inflow takes no more than 10 seconds to perform. This advantage is especially important as many patients with acute type-A aortic dissection are haemodynamically unstable. In our series, over 40% of patients had arrested while opening the chest such that CPB had to be established expeditiously. There were no deaths in these patients.

The technique also allows cannulation in patients which would have otherwise no other available sites for establishing arterial inflow. These include patients with extensive dissections flaps extending to the subclavian vessels and femoral arteries. We recently operated on a 50-year old man with dissection flaps in all his neck vessels with subtotal occlusion of all three neck arteries. The subclavian artery was also involved and the dissection extending well beyond the femoral vessels. The only available site for cannulation was the left ventricular apex and this was achieved without incident.

We believe that the transapical technique provides a more physiological method of delivering arterial inflow in that it assures perfusion of the true lumen. The monitoring and modification of flow rate by the perfusionist along with real time imaging on TOE allows careful control of antegrade flow which is important in the presence of multiple dissection flaps.

In our opinion surgeons should be aware of this technique and its advantages in current cardiac practice. This is our method of choice of cannulation for type-A dissection repair in the absence of a stenotic aortic valve that would preclude the passage of the cannula across it.

## Competing interests

The author(s) declare that they have no competing interests.

## Authors' contributions

AWS performed all the operations with this technique, conceived of the article and critically revised the manuscript. RSJ also conceived of the article and was responsible for its drafting. NM was responsible for data collection and helped in drafting the manuscript. CA and JS were responsible for providing important intellectual content throughout the manuscript's production and for approval of the final version. All authors read and approved the final manuscript.

## References

[B1] Neri E, Massetti M, Capannini G (1999). Axillary artery cannulation in type a aortic dissection operations. J Thorac Cardiovasc Surg.

[B2] Banbury MK, Cosgrove DM (2000). Arterial cannulation of the innominate artery. Ann Thorac Surg.

[B3] Zwart HH, Kralios A, Collan R, Kolff WJ (1969). Transarterial closed-chest left ventricular (TaCLV) bypass. Trans Am Soc Artif Intern Organs.

[B4] Golding LA (1985). New cannulation technique for the severely calcified ascending aorta. J Thorac Cardiovasc Surg.

[B5] Robicsek F (1991). Apical aortic cannulation: application of an old method with new paraphernalia. Ann Thorac Surg.

[B6] Wada S, Yamamoto S, Honda J, Hiramoto A, Wada H, Hosoda Y (2006). Transapical aortic cannulation for cardiopulmonary bypass in type A aortic dissection operations. J Thorac Cardiovasc Surg.

[B7] Jutley RS, Masala N, Sosnowski AW (2007). Transapical aortic cannulation: the technique of choice for Type A dissection. J Thorac Cardiovasc Surg.

